# Maternal immunization with ovalbumin or *Dermatophagoides pteronyssinus* has opposing effects on FcγRIIb expression on offspring B cells

**DOI:** 10.1186/1710-1492-10-47

**Published:** 2014-09-02

**Authors:** Aline Aparecida de Lima Lira, Marília Garcia de Oliveira, Luana Mendonça de Oliveira, Alberto José da Silva Duarte, Maria Notomi Sato, Jefferson Russo Victor

**Affiliations:** Laboratory of Dermatology and Immunodeficiencies, LIM-56, University of São Paulo, Medical School, Av. Dr. Enéas de Carvalho Aguiar, 500, 3rd floor, 05403-000 São Paulo, Brazil

**Keywords:** Allergy, Maternal immunization, *Dermatophagoides pteronyssinus*, FcγRIIb

## Abstract

**Background:**

Over the last decade, our group has demonstrated that murine preconception immunization with allergens has a protective effect on allergy development in offspring. The murine model used in the present study allowed us to compare allergy induction by ovalbumin (OVA) and dust mite extract from *Dermatophagoides pteronyssinus* (Dp).

**Findings:**

Female mice were immunized with OVA or Dp. Pups from immunized and non-immune mothers were immunized at 3 days old (do) with the same antigen used for the maternal immunization. The offspring were analyzed at 20 do. Preconceptional immunization with OVA or Dp did not increase maternal IgE serum levels, although the immunizations induced an increase in allergen-specific IgG1 Ab levels. Offspring serum analyses revealed that maternal immunization with OVA suppressed IgE production only in offspring immunized with OVA. Both preconception immunization protocols inhibited cellular influx into the airways of immunized offspring compared with controls. Similar frequencies of offspring IgM + B cells were found in the OVA- and Dp-immunized groups compared with their respective control groups. Moreover, preconception immunization with OVA enhanced FcγRIIb expression on OVA-immunized offspring B cells. In contrast, decreased FcγRIIb expression was detected on Dp-immunized offspring B cells compared with cells from the offspring of non-immune mothers.

**Conclusions:**

Together, these results show that preconception OVA immunization and Dp immunization can inhibit allergy development but have opposite effects on FcγRIIb expression on offspring B cells.

## Introduction

In an earlier study, we showed that preconception immunization with the mite *Dermatophagoides pteronyssinus* (Dp) could suppress anaphylactic IgE production and regulate Th2 cytokine exacerbation in offspring [1]. Subsequently, we observed that preconception immunization with ovalbumin (OVA) could induce the passive transference of maternal anti-OVA IgG1 antibodies (Abs) at high levels [2], which could be detected in the sera of offspring concomitant with the increased expression of inhibitory FcγRIIb receptors on B cells [3]. Co-localizing with B cell receptors (BCRs), FcγRIIb receptors can interact with IgG/antigen immune complexes and phosphorylate the inhibitory phosphatase SHIP, leading to the inhibition of B cell activation [4]. This process hinders formation of the immunological synapse between B cells and CD4+ T cells, which is necessary for isotype switching and IgE production [5].

Our hypothesis was that maternal antibodies (MatAbs) transferred during pregnancy and breastfeeding can form immune complexes with allergens from offspring and inhibit the activation of offspring B cells. In humans, the increased passive transfer of anti-Dp MatAbs does not have a protective effect on offspring [6]. Although OVA is more widely used in murine models of type I hypersensitivity, Dp is an important commonly inhaled allergen that causes bronchial asthma and allergic rhinitis in humans [7]. To date, the effect of maternal immunization with Dp on the expression of inhibitory receptors in offspring B cells has not been evaluated. In this study, we use a murine model to compare the effects of OVA and Dp immunization to better understand neonatal allergy regulation and to guide future studies of allergy regulation in humans.

## Methods

### Mice

Male and female C57BL/6 inbred mice were used at 8 to 10 weeks of age. Animals were purchased from the Central Animal Facility of the School of Medicine, University of Sao Paulo. Offspring were used during the neonatal period (3 days old (do)). All experiments described n this manuscript were approved by the University of Sao Paulo – School of Medicine - Animal Ethics Committee (CEP-FMUSP: 097/11 - Sao Paulo, SP, Brazil).

### Immunization protocols

Female mice were immunized subcutaneously with 1500 μg OVA (Sigma, USA) or 10 μg Dp (Indoor Biotechnologies, USA) in 6 mg Alum (FURP, Sao Paulo) and boosted after 10 and 20 days with 1000 μg OVA or 10 μg Dp in saline intraperitoneally (i.p.). Females were mated at 21 days post immunization. The pups of immunized and non-immune mothers were immunized with the same antigen used for maternal immunization. Offspring at 3 do were immunized (i.p.) with 100 μg OVA or 10 μg Dp in 0.6 mg Alum and boosted after 10 days with the same antigen/dose in saline. Sera from the mothers were obtained at term. Experimental analyses of the offspring were performed at 20 do. As a control group, non-immunized offspring from non-immune mothers were bled at 20 do, and the sera were analyzed for total IgE production.

### Determination of total IgE and anti-OVA/Dp IgG1 Ab levels

OVA- and Dp-specific IgG1 and total IgE antibodies were measured by ELISA, as previously described [2]. To measure total IgE, a standard curve was used (Pharmingen, USA). The anti-OVA and anti-Dp Ab levels are expressed as optical densities.

### Lung inflammation

Offspring from either immune or non-immune mothers were immunized and subjected to nasal instillations with 100 μg OVA or 10 μg Dp at 43, 50, 57, 58 and 59 do. Bronchoalveolar fluid (BAL) was analyzed at 60 do following exsanguination of the abdominal aorta. The BAL was obtained by washing the lungs with three times with 1.5 mL PBS using a tracheal tube, which was then centrifuged at 800 rpm for 10 min. The cell pellet was diluted in 300 μL PBS, and total leukocyte counts were performed using a Neubauer chamber.

### Spleen cell suspension

Spleens were collected, and cells were harvested for flow cytometric analysis, as previously described [3].

### Flow cytometry

For surface staining, a single-cell suspension in PBS + 1% BSA was incubated with the following fluorescent-conjugated Abs (BD Biosciences, USA): anti-CD19, anti-CD40, anti-IgM or anti-CD16/32. Gating of the cells was based on specific isotype control values. Data were acquired using an LSRFortessa cytometer (BD Biosciences, USA) and analyzed with the FlowJo software program (Tree Star, USA).

### Statistical analysis

Differences between groups were considered significant when *P* values were < 0.05 according to the *Mann–Whitney* test.

## Findings

**Figure 1:**
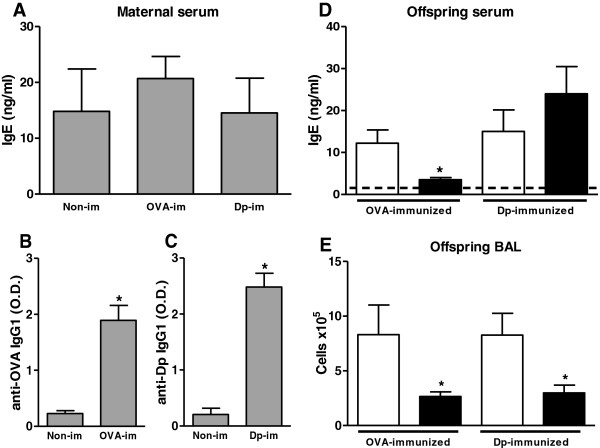
**Decreased pulmonary inflammation in offspring in response to preconception OVA or Dp immunization.** Female mice were immunized with OVA or Dp, boosted 10 and 20 days later, and mated on day 21. Sera from full-term pregnancies were evaluated for **(A)** total IgE, **(B)** anti-OVA IgG1 and **(C)** anti-Dp IgG1 Ab levels by ELISA. Sera from full-term pregnancies of non-immune females were assessed as controls. Offspring at 3 do were immunized with the same antigen used for the maternal immunizations, boosted at 13 do, and bled at 20 do. The offspring of control non-immune mothers were immunized with OVA or Dp as controls. Total IgE levels were determined by ELISA, and the dashed line represents IgE levels in control non-immunized 20 do offspring from nom-immune mothers **(D)**. At 43 do, the offspring received five intranasal challenges with OVA or Dp. After 24 h, BAL was assessed, and total cell counts were evaluated **(E)**. The data are presented as mean (8 to 10 mice per group) ± SEM values. *P ≤ 0.05 compared with the control group.

First, we assessed maternal serum Ab levels at full-term pregnancy after preconception immunization with OVA or Dp. Preconception immunization with OVA or Dp did not increase serum IgE levels (Figure 1A), although the immunizations did increase the levels of allergen-specific IgG1 Abs (Figures 1B-C). All immunized groups produced higher levels of total IgE compared with control non-immune offspring from non-immune mothers (Figure 1D). Analysis of the sera from the immunized offspring revealed that maternal immunization with OVA suppressed IgE production only in offspring immunized with OVA (Figure 1D) but not with Dp (Figure 1D).

Moreover, we evaluated the effect of maternal allergen immunization on pulmonary inflammation in offspring, and we verified that both preconception immunization protocols inhibited cellular influx into the airways of immunized offspring compared with offspring from non-immune mothers (Figure 1E). 

**Figure 2:**
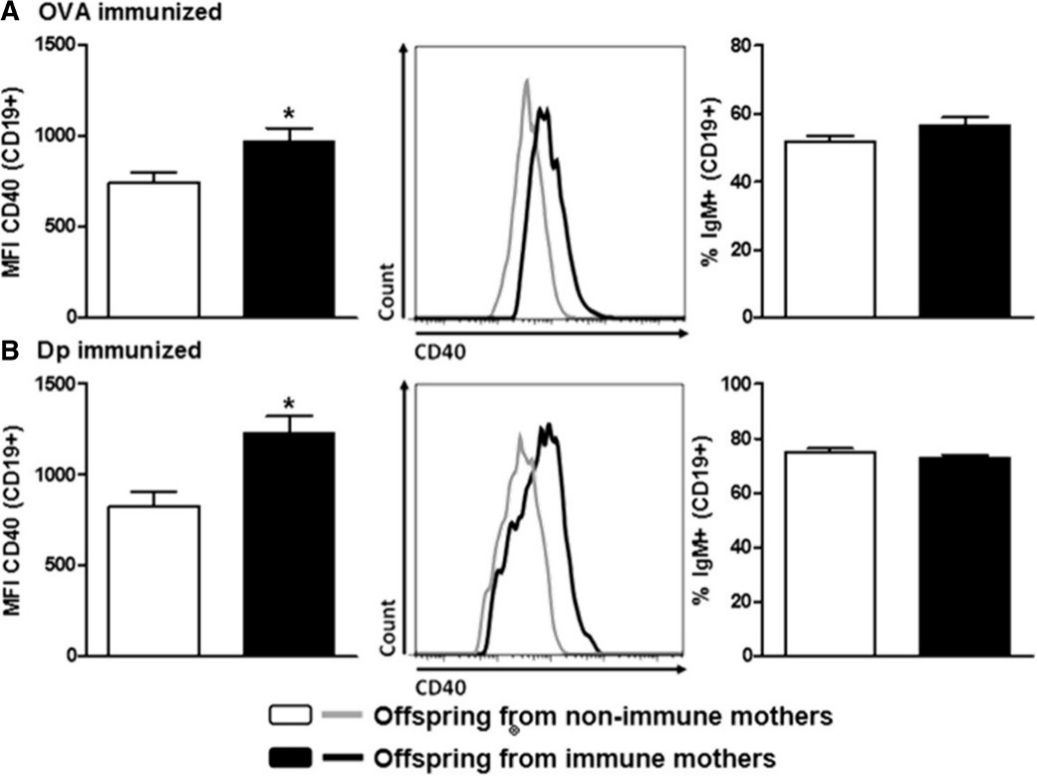
**Increased CD40 expression on offspring B cells induced by maternal immunization.** Offspring from **(A)** OVA- or **(B)** Dp-immunized mothers were immunized at 3 do with the same antigen used in the maternal immunization and boosted at 13 do. At 20 do, splenic CD19+ B cells were assessed for CD40 expression and the percentage of CD19+ IgM + cells. The control group was composed of offspring from non-immune mothers immunized with OVA or Dp. Representative histograms of CD40 expression for each group are shown (**A**-**B**, middle panels). The data are presented as mean (8 to 10 mice per group) ± SEM values. *P ≤ 0.05 compared with the control group.

**Figure 3:**
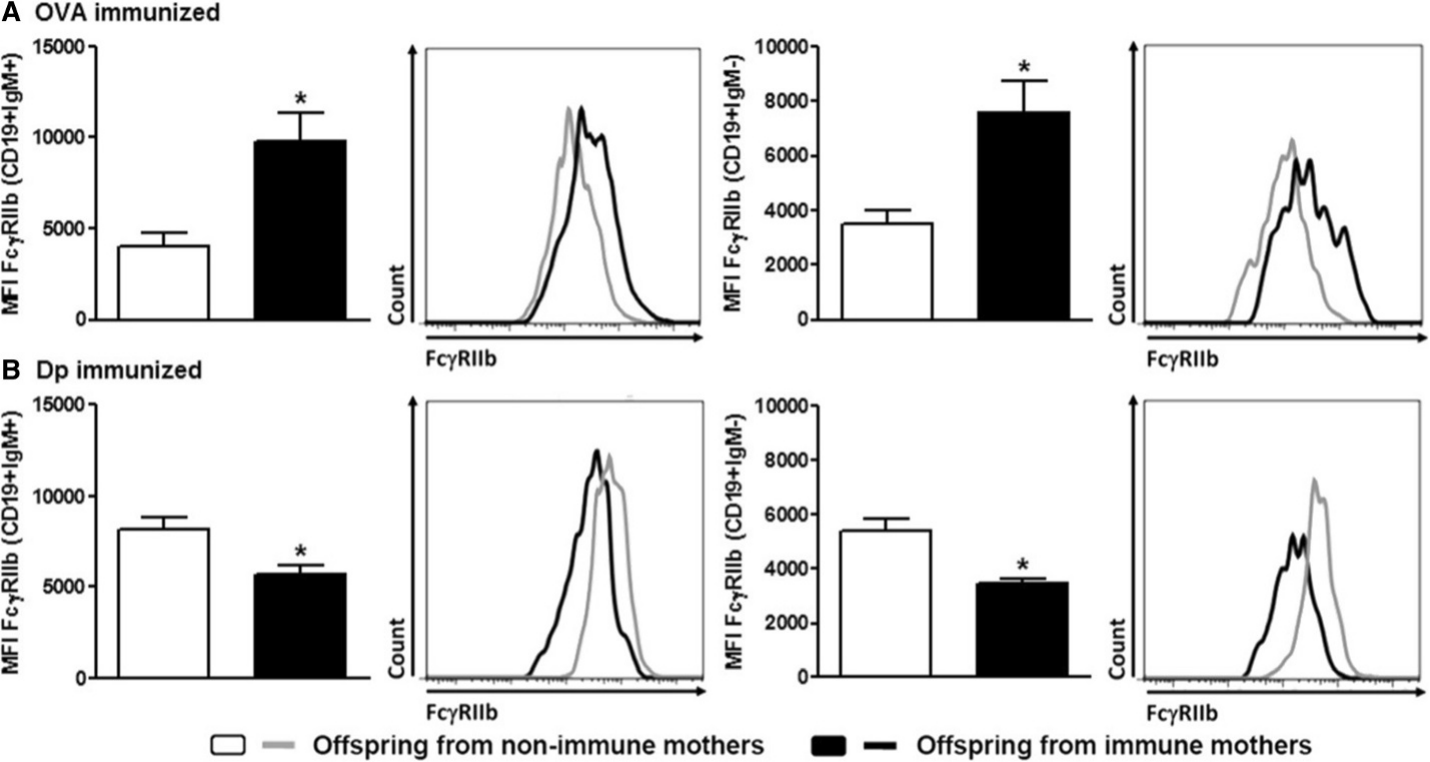
**The maternal antigen immunization protocols yield opposing results with respect to FcγRIIb expression on offspring B cells.** Offspring from **(A)** OVA- or **(B)** Dp-immunized mothers were immunized at 3 do with the same antigen used for the maternal immunization and boosted at 13 do. At 20 do, splenic CD19+ B cells were evaluated for FcγRIIb expression. The control group was composed of offspring from non-immune mothers immunized with OVA or Dp. Representative histograms of FcγRIIb expression in each group are shown on the right side of the respective graph. The data are presented as mean (8 to 10 mice per group) ± SEM values. *P ≤ 0.05 compared with the control group.

To verify the effect of maternal immunization on offspring B cells, we evaluated CD40 and FcγRIIb expression on these cells. We observed that both maternal immunization protocols could upregulate CD40 expression on offspring B cells compared with B cells from the control offspring groups (Figure 2A). Similar frequencies of offspring IgM + B cells were found in the OVA- and Dp-immunized groups compared with their respective control groups (Figure 2B). Moreover, preconception immunization with OVA enhanced FcγRIIb expression on OVA-immunized offspring B cells compared with B cells from OVA-immunized offspring from non-immune mothers (Figure 3A). In contrast, decreased FcγRIIb expression was observed on Dp-immunized offspring B cells compared with B cells from the Dp-immunized offspring control group (Figure 3B).

## Discussion

By varying the allergen dose in the maternal immunization protocol from 1 to 10 μg Dp, we were able to detect an increase in the maternal Ab response and passive Ab transference in the highest titers (10 μg) (data not shown). This dose could also inhibit the allergic response and lung inflammation in offspring and was therefore adopted as the standard dose.

Neither preconception immunization protocol induced higher total IgE levels, suggesting that the females did not reach allergic status and that both protocols could sensitize the maternal immune system. As we previously described, MatAbs are passively transferred to their offspring, concomitant with anaphylactic IgE suppression in offspring [1–3]. Preconception OVA immunization could reduce total IgE production in offspring, in contrast with maternal Dp immunization. However, both protocols could downregulate pulmonary inflammation in offspring, suggesting that allergy development in the offspring was inhibited by preconception OVA or Dp immunization.

Phenotypic analysis of offspring B cells revealed an equivalent increase in CD40 expression on offspring B cells from both types of immune mothers. However, this status could not have influenced isotype switching of the offspring B cells in response to neonatal immunization, as the percentages of IgM + B cells were similar between pups from OVA- or DP-immunized mothers compared with those from non-immune mothers.

Maternal OVA immunization can induce the upregulation of FcγRIIb receptors on offspring B cells at 20 do, as described previously by our group [3]. The inhibitory co-receptor FcγRIIb can inhibit the *in vitro* activation of B cells [4, 8, 9] and induce apoptosis in plasma cells [10]. Therefore, the upregulation of FcγRIIb levels on B cells in the presence of immune complexes may represent an effective way to downregulate immune activation. We detected the overexpression of FcγRIIb on offspring IgM + B cells, which consisted primarily of naïve B cells. This result is consistent with our hypothesis that passively transferred MatAbs can negatively regulate offspring B cells by interacting with allergens in the offspring and inhibiting offspring B cell activation and isotype switching, thereby downregulating IgE production and allergy development. Our results demonstrate that this effect cannot be induced by maternal Dp immunization, for which an opposite effect was observed, with offspring B cells showing downregulated FcγRIIb expression in response to maternal Dp immunization.

Our findings suggest that maternal Dp immunization does control offspring allergy development but without the upregulation of FcγRIIb on offspring B cells, as observed in the maternal OVA immunization protocol [3]. This phenomenon could be due to the fact that the Dp extract contains a variety of proteins with biological activity, in contrast with purified OVA. The main antigenic fraction of Dp (Der p 1) is a 25 kDa cysteine protease. Recombinant Der p 1 and natural Der p 1 have similar biological effects on immune cells [11]. These effects include the optimization of antigen capture and processing by APCs [12], as well as the cleavage of CD23 on B cells [13, 14] and of CD25 on T cells [15]. This activity blocks the negative feedback mediated by IgE Abs that interact with B cells via CD23 and the induction of low IFN-γ and high IL-4 production due to CD25 cleavage on CD4+ T cells [16].

The regulation of FcγRIIb expression on B cells in response to CD23 activity has not been described, although it is possible that Dp antigenic activity may indirectly influence FcγRIIb expression. The fact that maternal Dp immunization can inhibit pulmonary inflammation in offspring, independent of FcγRIIb upregulation on offspring B cells, may be due to other mechanisms, such as allergen neutralization and idiotypic interactions [17] or the induction of offspring regulatory T cells [18]. In any case, the activity of these mechanisms appears to result in a similar mode of regulation in offspring until 20 do in mice, as we show in this report. Future studies on preconception Dp immunization should examine purified Dp proteins with similar structural and biological activities as the Dp extract.
